# Validation of the recording of idiopathic pulmonary fibrosis in routinely collected electronic healthcare records in England

**DOI:** 10.1186/s12890-023-02550-0

**Published:** 2023-07-11

**Authors:** Ann Morgan, Rikisha Shah Gupta, Peter M. George, Jennifer K. Quint

**Affiliations:** 1 School of Public Health, Imperial College London, Level 9, Sir Michael Uren Hub, White City Campus, 86 Wood Lane, W12 0BZ London, UK; 2 National Heart and Lung Institute, Imperial College London, Level 9, Sir Michael Uren Hub, White City Campus, 86 Wood Lane, W12 0BZ London, UK; 3 Interstitial Lung Disease Unit, Royal Brompton and Harefield NHS Foundation Trust, London, UK; 4 NIHR Imperial Biomedical Research Centre, The Bays, Entrance, 2 S Wharf Rd, W2 1NY London, UK

**Keywords:** Interstitial lung disease, Idiopathic pulmonary fibrosis, Pulmonary fibrosis, Validation, CPRD, HES, Diagnostic codes

## Abstract

**Background:**

Routinely-collected healthcare data provide a valuable resource for epidemiological research. Validation studies have shown that for most conditions, simple lists of clinical codes can reliably be used for case finding in primary care, however, studies exploring the robustness of this approach are lacking for diseases such as idiopathic pulmonary fibrosis (IPF) which are largely managed in secondary care.

**Method:**

Using the UK’s Clinical Practice Research Datalink (CPRD) Aurum dataset, which comprises patient-level primary care records linked to national hospital admissions and cause-of-death data, we compared the positive predictive value (PPV) of eight diagnostic algorithms. Algorithms were developed based on the literature and IPF diagnostic guidelines using combinations of clinical codes in primary and secondary care (SNOMED-CT or ICD-10) with/without additional information. The positive predictive value (PPV) was estimated for each algorithm using the death record as the gold standard. Utilization of the reviewed codes across the study period was observed to evaluate any change in coding practices over time.

**Result:**

A total of 17,559 individuals had a least one record indicative of IPF in one or more of our three linked datasets between 2008 and 2018. The PPV of case-finding algorithms based on clinical codes alone ranged from 64.4% (95%CI:63.3–65.3) for a “broad” codeset to 74.9% (95%CI:72.8–76.9) for a “narrow” codeset comprising highly-specific codes. Adding confirmatory evidence, such as a CT scan, increased the PPV of our narrow code-based algorithm to 79.2% (95%CI:76.4–81.8) but reduced the sensitivity to under 10%. Adding evidence of hospitalisation to the standalone code-based algorithms also improved PPV, (PPV = 78.4 vs. 64.4%; sensitivity = 53.5% vs. 38.1%). IPF coding practices changed over time, with the increased use of specific IPF codes.

**Conclusion:**

High diagnostic validity was achieved by using a restricted set of IPF codes. While adding confirmatory evidence increased diagnostic accuracy, the benefits of this approach need to be weighed against the inevitable loss of sample size and convenience. We would recommend use of an algorithm based on a broader IPF code set coupled with evidence of hospitalisation.

**Supplementary Information:**

The online version contains supplementary material available at 10.1186/s12890-023-02550-0.

## Background

Idiopathic pulmonary fibrosis (IPF) is characterised by worsening respiratory symptoms (dry cough, exertional dyspnoea) and irreversible loss of lung function due to progressive architectural distortion and aberrant cellular proliferation within the interstitium of the lung. The prognosis for people diagnosed with this chronic lung condition is poor, with a median survival of only 3–5 years if left untreated [1–3]. Global incidence and prevalence is estimated to be in the range 0.09–1.30 and 0.33–4.51 per 10,000 persons respectively, but there is wide variation in the estimates reported by individual countries [4, 5]. While IPF remains a relatively rare disease, there is some evidence to suggest that prevalence is increasing [6]. However, it is unclear whether this is due to increased recognition, changes in disease nomenclature and classification, or a true increase [5].

Analysis of primary care data for 2000–2012 suggests a UK IPF prevalence at the higher end of global estimates, between 19.9 and 38.8 cases per 100,000 people, with approximately 5,000 new cases diagnosed each year (incidence 2.8–8.6 per 100,000 person-years) [7]. Despite increased interest in IPF and other interstitial lung diseases (ILDs) with a similarly progressive nature, more recent and robust estimates of UK prevalence and incidence remain elusive. This can be attributed, at least in part, to uncertainties and difficulties in obtaining reliable, representative IPF case numbers from routinely-collected health data, which include delays in making a clinical diagnosis of IPF and likely changes in the way healthcare professionals have recorded and coded diagnoses of IPF [8] over the past decade in the wake of revised ATS/ERS guidelines on IPF diagnosis and management in 2011 and again in 2018 [1, 9].

While routinely-collected healthcare data represent a valuable resource for epidemiological study and research, their utility relies on the quality of case ascertainment. Validation studies are important to assess the reliability of diagnostic coding of diseases and the majority of validation studies conducted to date indicate that case ascertainment based on the use of simple lists of clinical diagnostic codes in primary care records results in acceptable positive predictive values (PPVs) for the majority of diseases and conditions, especially those that are predominantly managed in primary care [10]. However, studies of this type have not been widely conducted for IPF. To our knowledge, only one such study has been performed, and this was limited to just one code – cryptogenic fibrosing alveolitis, a historic term for IPF [11]. Moreover, this study was published in 2000 and predates the transition to medical coding systems that are based on SNOMED CT terminology and codes.

The aim of this study was to assess the reliability of recording IPF in a UK primary care database. Specifically, to determine whether a list of clinical diagnostic codes alone was sufficient or whether a more complex algorithm – one that includes additional information such as radiology or hospital admission data is required to improve the accuracy of IPF case identification in primary care data. To do this, we used ONS mortality data as our reference or “gold standard”, relying on the assumption that a person with IPF on their death certificate is highly likely to have been diagnosed with IPF during their lifetime.

## Methods

### Data sources

We used data from the Clinical Practice Research Datalink (CPRD) Aurum database (November 2020 build). This dataset contains pseudonymized primary care electronic health records for nearly 40 million patients, including around 13 million current patients representing 19% of the UK population [12]. CPRD Aurum captures data from participating GP practices using the EMIS web patient management software and provides longitudinal patient-level information (from patients’ date of registration) on demographic and selected lifestyle characteristics, symptoms and clinical diagnoses, vaccination history, laboratory test results, prescriptions and referrals to secondary care. Information is recorded by practice staff using a combination of SNOMED CT (UK edition), Read (version 2) and local EMIS Web codes (Table 1). The free-text part of patients’ primary care records is not currently made available for observational research purposes. CPRD Aurum data have been shown to be representative of the UK population in terms of geographical distribution, as well as age and gender [13].

**Table 1:** Data sources

Data set	Coverage	Key information (including coding system)
***Primary care***		
CPRD Aurum	Start of patient GP registration – c. October 2020	Symptoms and clinical diagnoses, demographic characteristics, vaccination history, lifestyle and behaviours (e.g. smoking history), laboratory test results, prescriptions, referrals to secondary care (SNOmed codes, READ codes)
***Linked data sets***		
ONS Death registration data	1998–2020	Date, place and cause of death including underlying and contributory causes of death (ICD-10)
Hospital Episode Statistics (HES)		
Admitted Patient Care (APC)	1997–2020	Details of all admissions to NHS hospitals in England (including acute, mental and primary care trust hospitals) including clinical diagnoses (ICD-10) and procedures performed (OPCS)
Diagnostic Imaging Dataset (DID)	2012–2020	Type of imaging performed (CT, X-ray, MRI) and body area imaged (e.g. chest)

CPRD Aurum primary care data are routinely linked to a number of other patient-level datasets, including Hospital Episode Statistics (HES) and ONS mortality data using a deterministic methodology [14]. Linked data are only available for patients registered at GP practices in England that have consented to participate in the linkage scheme (c. 70% of English practices). For the purposes of this study, we made use of linkages to HES Admitted Patient Care (APC) data, the HES Diagnostic Imaging Dataset (DID), and ONS Death Registration Data. HES APC provides data on all admissions to NHS hospitals in England [15]. The HES Diagnostic Imaging Dataset (DID) includes data on the type of diagnostic imaging performed and body region, extracted from NHS radiological information systems. The ONS dataset contains information on the date, place and causes of death (underlying and contributory) extracted from death certificates for all deaths registered in England and Wales. Both HES APC data and ONS cause-of-data death are coded using ICD-10 codes (Table 1).

### Study design and population

This analysis uses a cohort study design. The study population was drawn from patients registered at English CPRD practices that had consented to HES/ONS linkage. To be eligible for inclusion in the study patients had to have evidence of a diagnosis of IPF in one or more of our three linked electronic health datasets, CPRD Aurum, HES APC or ONS, and be aged at least 18 years at the start of the study period (1 January 2008–31 December 2018) and prior to their IPF diagnosis. A period of “useable” follow up (1 day) was defined for each patient based in their primary care record, starting on the latest of 1 January 2008, date of IPF diagnosis (the earliest IPF code in Aurum), start of current registration at a linked GP practice or date of 18th birthday and ending on 31 December 2018, date of death, last day of data collection at the practice or patient’s transfer out date, whichever came first.

### Case definitions

#### CPRD Aurum

A set of clinical SNOMED CT diagnostic codes denoting IPF created for this study was used to define cases. This code set was developed using an established methodology (https://github.com/NHLI-Respiratory-Epi/SNOMED-CT-codelists). In short, initial text-based search terms were derived in consultation with two respiratory clinicians with expert knowledge of ILDs. Codes found by searching of the Aurum medical browser using these search terms were cross-checked against published IPF code lists, and any codes not captured by the test-based search added to form a list of potential codes [7, 8, 11, 16]. These codes were then independently rated by the same clinical experts as “yes” for the codes which were strongly indicative of an IPF diagnosis, “maybe” for codes that conceivably might suggest IPF (to allow for variability in coding practices) and “no” for highly unlikely that a person has IPF. Codes rated as “no” were rejected and not used further in this analysis.

Consistent with previous electronic health records-based studies that have used sets of clinical codes to define IPF cohorts, [7, 8, 16] and based on our “yes” and “maybe” rated list, we created two separate code lists, one comprising a set of more focused, narrowly-specified codes, strongly indicative of an IPF diagnosis, and the other a set of more generic, less specific codes denoting a diagnosis of pulmonary fibrosis of any aetiology, not necessarily IPF (see Table S1; https://github.com/NHLI-Respiratory-Epi/Validation-of-the-recording-of-Idiopathic-Pulmonary-Fibrosis-in-routinely-collected-electronic-healt). We defined two IPF primary care cohorts, one based on the “broad” set of Aurum codes, the other on the “narrow” Aurum code set.

#### HES/ONS data

We used the International Classification of Diseases, 10th revision (ICD-10) codes, J84.1, J84.8 and J84.9 (https://github.com/NHLI-Respiratory-Epi/Validation-of-the-recording-of-Idiopathic-Pulmonary-Fibrosis-in-routinely-collected-electronic-healt) to define IPF hospitalizations and deaths in HES APC and ONS mortality data, respectively. To be included in the HES APC cohort, a patient had to have one of the three qualifying ICD-10 codes recorded as either the first or second clinical diagnosis in any “episode” of hospital care within their stay (“spell”) in hospital within their follow up period. We chose to restrict our case definitions to the first and second diagnostic codes in order to be more certain that we were selecting patients where IPF was the reason for their admission. In HES-APC data, codes mentioned further down the list of recorded diagnoses are more likely to reflect coexisting conditions/comorbidities that might impact hospital care and are known to have lower diagnostic accuracy. In a sensitivity analysis, we also created a cohort which included patients who had a least one qualifying ICD-10 IPF code in any diagnostic position (up to 20 are allowed).

Patients who had either J84.1, J84.8 or J84.9 listed as cause of death anywhere in their ONS record were assumed to have IPF and formed the basis of our gold standard population. For sensitivity analysis purposes, we also created an ONS population comprised of individuals who had IPF recorded as their underlying cause of death.

#### Risk factors and comorbidities

For patients who had evidence of IPF in CPRD Aurum and/or HES APC, we assessed the prevalence of various risk factors (age, sex and smoking history) and comorbidities (COPD, asthma, lung cancer, ischemic stroke, heart failure, ischemic heart disease, myocardial infarction, gastroesophageal reflux) as recorded in primary care. Smoking status was based on the closest available record to the date of IPF diagnosis; patients were assumed to have a given comorbidity if they had a relevant diagnostic code at any point prior to their IPF diagnosis. All codelists are available via GitHub (Validation-of-the-recording-of-Idiopathic-Pulmonary-Fibrosis-in-routinely-collected-electronic-healt/broad_and_narrow_ipf-aurum_snomed_read.tsv ).

### Analysis

#### Descriptive analysis

To gain insight into possible changes in coding practices in primacy care over time, we examined the frequency of use of IPF codes in Aurum for each year of our study period (2008–2018).

#### Concordance in the recording of IPF across Aurum, HES-APC and ONS

We totalled the number of patients who met our cohort inclusion criteria in each of our three concurrent linked data sets using the case definitions described above. To assess concordance, the Aurum and HES-APC cohorts were restricted to those who had died within our study period. We investigated the concordance of recording of IPF in ONS, Aurum and HES-APC in terms of the number of patients with at least one IPF record in (i) just Aurum, (ii) just HES-APC and (iii) just ONS, (iv) both Aurum and HES-APC, (v) Aurum and ONS, (vi) HES-APC and ONS, and (vii) Aurum, HES-APC and ONS.

#### Diagnostic algorithms and validation

In total we defined eight diagnostic algorithms (DAs) based on either our “broad” or “narrow” clinical code sets plus any one or more of the following: evidence of HRCT (high-resolution computed tomography) of the thorax, absence of other known causes of interstitial lung disease (such as connective tissue disease, sarcoidosis, domestic and occupational environmental exposures, drug toxicity and other non-IPF ILDs), and/or evidence of hospitalisation for IPF, i.e. record in HES-APC (Table 2).

**Table 2:** Code-based diagnostic algorithms

Diagnostic algorithm	Data source (Aurum and/or HES)	Description
DA1	Aurum	• Any broad code in Aurum
DA2	Aurum and HES DID	• Any broad code in Aurum and • No other known causes of ILD recorded in Aurum^a^ and • Evidence of HRCT of thorax at any time in HES DID
DA3	Aurum	• Any narrow code in Aurum
DA4	Aurum and HES DID	• Any narrow code in Aurum and • No other known causes of ILD recorded in Aurum^a^ and • Evidence of HRCT of thorax at any time in HES DID
DA5	HES-APC	• ICD-10 codes J84.1, J84.8 or J84.9 as primary or secondary discharge diagnosis in any “episode” within a hospital spell
DA6	HES-APC and HES DID	• ICD-10 codes J84.1, J84.8 or J84.9 as primary or secondary discharge diagnosis in any “episode” within a hospital spell and • Evidence of HRCT of thorax at any time
DA7	Aurum and HES-APC	• Any broad code in Aurum and • ICD-10 codes J84.1, J84.8 or J84.9 as primary or secondary discharge diagnosis in any s “episodes” within a hospital spell
DA8	Aurum and HES-APC	• Any narrow code in Aurum and • ICD-10 codes J84.1, J84.8 or J84.9 as primary or secondary discharge diagnosis in any “episode” within a hospital spell

*DA *Diagnostic algorithm, *IPF *Idiopathic pulmonary fibrosis, *ILD *Interstitial lung disease, *HRCT *High resolution computed tomography, *CPRD *Clinical Practice Research Datalink, *HES *Hospital Episode Statistics

^a^Connective tissue disease, sarcoidosis, domestic and occupational environmental exposures, drug toxicity and other non-IPF ILD

PPV was calculated as the number of IPF cases correctly identified by the algorithm (i.e. confirmed by a record in ONS) divided by the total number of IPF cases identified by that algorithm. Sensitivity was defined as the number of “true” IPF cases correctly identified by the algorithm divided by the total number of ONS IPF deaths. Given that a death is our reference standard, this analysis was limited to those patients who died.

All analyses were conducted using Stata version 17.0.

## Results

### Coding practices in primary care over time

**Fig. 1:**
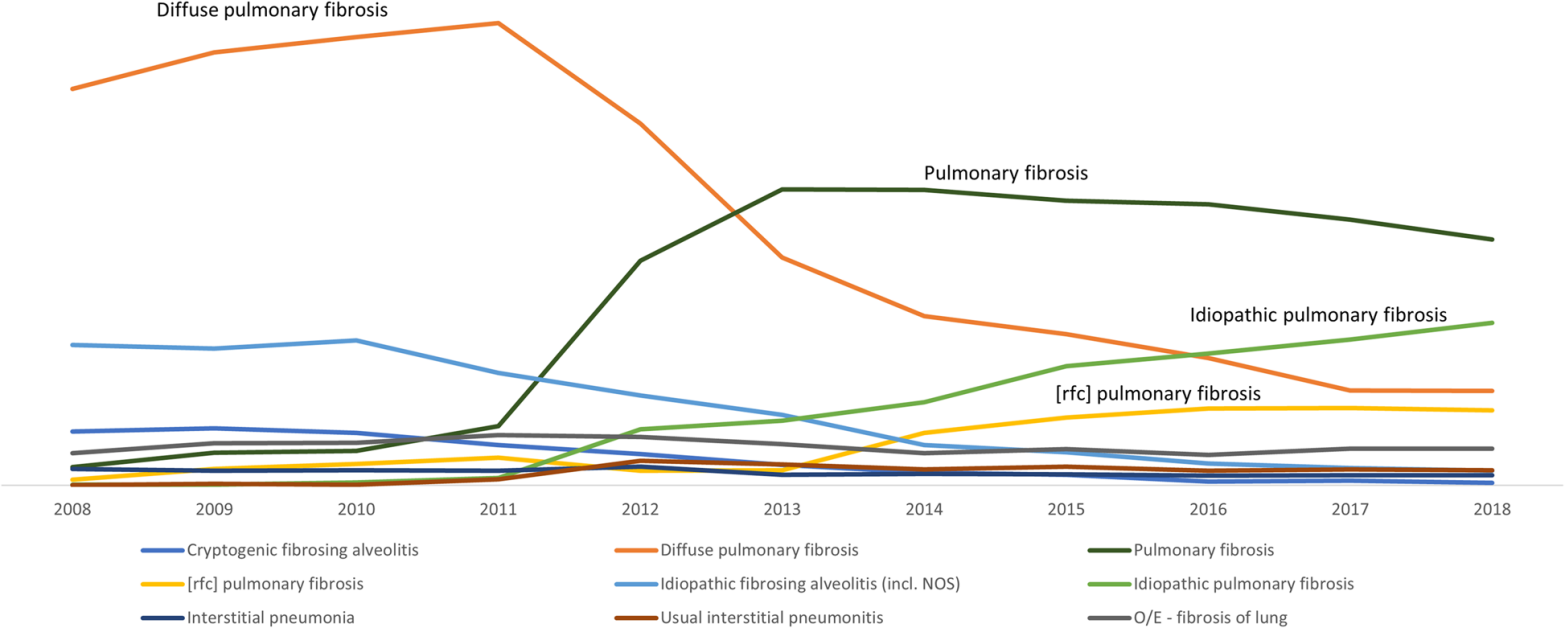
Trends in the use of selected IPF clinical codes in CPRD Aurum primary care data, 2008–2018

Between 2008 and 2018, the most frequently used coding terms were; diffuse pulmonary fibrosis, pulmonary fibrosis, idiopathic fibrosing alveolitis and idiopathic pulmonary fibrosis. These four codes combined accounted for nearly 80% of all GP-coded IPF consultations during this period (Table S2). We also observed changes in the frequency of use of individual codes; for instance, there was decline in the use of terms such as ‘fibrosing alveolitis’ and ‘diffuse pulmonary fibrosis ’, but a marked increase in the utilization of codes associated with the terms ‘idiopathic pulmonary fibrosis’ and ‘pulmonary fibrosis’ (Fig. 1).

### Concordance

#### Study population

During the study period, 11,295 people died with IPF listed as a cause of death anywhere on their death certificate. The most frequently used ICD-10 code was J84.1, followed by J84.9. We found a total of 13,627 study-eligible adult patients who had at least one clinical code denoting a diagnosis of IPF in CPRD Aurum (based on a broad code set), of whom 9,498 died (of any cause). A slightly higher number, 14,719 had evidence of at least one hospital admission for IPF. Among this population, 10,714 people died. Our study population thus comprised a total of 17,559 individuals who died and had a IPF record in at least one of our three linked datasets (CPRD Aurum, HES-APC and ONS) within the time frame of our study. The patient attrition for the three sources is in Supplementary Fig. S1. The characteristics of our three base study cohorts are summarised in Table 3.

**Table 3:** Demographic and clinical characteristics of the study population (*n* = 17,557)

Characteristic	ONS % of total (n) unless otherwise stated	Aurum % of total (n) unless otherwise stated	HES-APC % of total (n) unless otherwise stated
Total cohort	**11,295 (100%)**	**9,948 (100%)**	**10,714 (100%)**
*Demographic*			
Median (IQR) age in years	77 (10.3)	77 (13)	77 (14)
≥ 65 years	90.0% (*n* = 10,176)	86.2% (*n* = 8,191)	85.3% (*n *= 9,145)
Male	63.0% (*n* = 7,123)	63.2% (*n* = 6,011)	60.7% (*n *= 6,512)
Smoking history (ever/current)	48.9% (*n* = 5,529)	66.2%(*n* = 6,288)	65.6% (*n *= 7,030)
*Comorbidities*			
COPD	18.2% (*n* = 2,060)	19.0% (*n* = 1,806)	20.9% (*n *= 2,240)
Asthma (in past 2 years)	6.93% (*n* = 784)	10.1% (*n* = 959)	10.3% (*n* = 1,111)
Lung cancer	3.0% (*n* = 348)	2.1% (*n* = 206)	2.8% (*n *= 305)
Ischemic stroke	13.4%(*n* = 1,513)	12.0% (*n* = 1,142)	13.2% (*n *= 1,418)
Heart failure	17.9% (*n* = 2,024)	17.1% (*n* = 1,629)	17.6% (*n *= 1,889)
Ischemic heart disease	13.4% (*n* = 1,523)	13.6% (*n* = 1,300)	13.4% (*n *= 1,441)
Myocardial infarction	13.1% (*n* = 1,481)	13.3% (*n* = 1,267)	13.2% (*n *= 1,414)
Gastroesophageal reflux	17.8% (*n* = 2,012)	17.6% (*n* = 1,677)	17.6% (*n* = 1,892)

#### ONS, Aurum and HES-APC

**Fig. 2:**
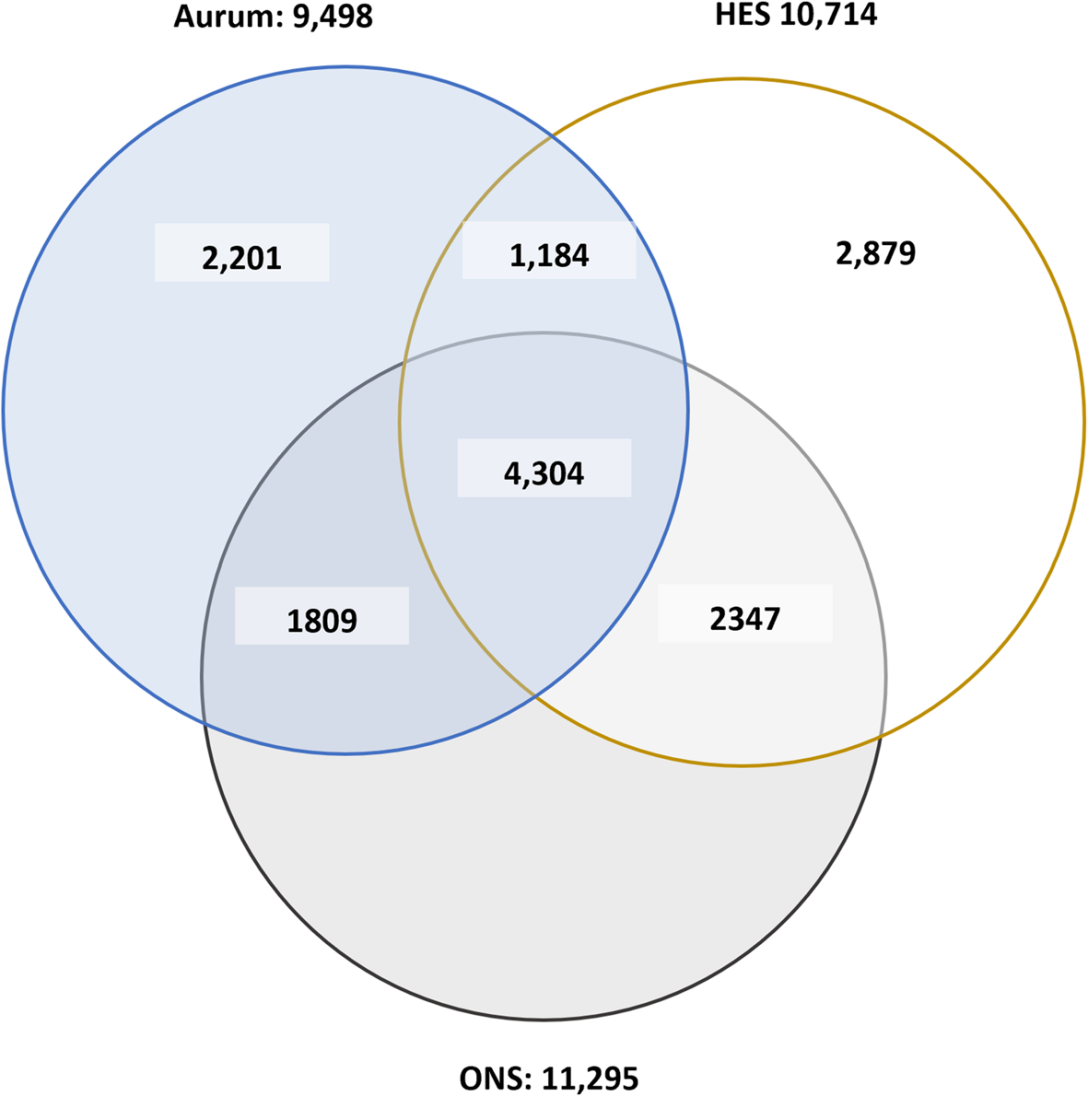
Concordance in the recording of IPF across ONS, CPRD Aurum and HES-APC. ONS, study eligible population with any cause of death (underlying or contributory) as IPF in their death record during the study period; Aurum, patients with one clinical code denoting IPF in Aurum (using a broad set of codes) and had died for any reason during the study period; HES-APC, patients admitted to the hospital for IPF at some point prior to their death for any reason (during the study period)

A quarter of individuals in our study population (24.5%, *n* = 4,304) had evidence of IPF in all three of our linked concurrent data sets (Fig. 2). Of the 11,295 adults who had evidence of IPF anywhere on the death certificate, 54.1% (*n *= 6,113) also had at least one clinical code denoting IPF in Aurum prior to death (or within 60 days after their death). Slightly more, 6,651 or 58.9% of the ONS population had been admitted to hospital ostensibly for IPF at some point prior to their death. Although only 38.1% (*n *= 4,304) of our ONS IPF population had evidence of an IPF diagnosis in both Aurum and HES-APC, 74.9% (*n *= 8,460) had IPF records in either Aurum or HES-APC (Fig. 2).

Conversely, of the Aurum IPF patients who died (*n* = 9,498), around a third (*n *= 3,385) had no mention of IPF on their death certificate. The most common underlying reason for death in these patients was pneumonia, bronchopneumonia or malignant neoplasm of unspecified part of bronchus or lung (ICD-10 codes J18.9, J18.0, C34.9, respectively). Other frequently reported causes were chronic obstructive pulmonary disease (COPD) (J44.9), “other general symptoms and signs” (R68.8), and “respiratory failure, not elsewhere classified” (J96.9). For those patients who were hospitalized with IPF but had no mention of IPF on their death certificate (*n *= 4,063), the top three underlying causes were again J18.9, J18.0 and C34.9; for this group other frequently reported causes included myocardial infarction, heart failure and sepsis (ICD-10 codes I29, I50.0, and A41.9, respectively).

#### Sensitivity analyses

Restricting our ONS reference population to those who had IPF listed as their underlying cause of death (*n *= 7,029) reduced the absolute number of patients in multiple datasets (Supplementary Fig. S2). The number of patients with records in both Aurum and ONS decreased from 6,113 to 4,159, or from 64.7 to 43.7% of the Aurum population (*n *= 9,498). Similarly, the number patients who were admitted to hospital with a diagnosis for IPF who also died of IPF, reduced to 4,756 or 44.4% of the HES-APC population (compared with 65.5% (*n *= 6,702) when the wider definition of the ONS population was used). Concordance across all three data sets also reduced when measured in terms of the absolute number of patients, from 4,304 to 3,119, which represents just 19.5% of the total study population (*n *= 15,957). On the basis of this analysis, which suggests that a higher proportion of Aurum and HES-APC cases are captured and “confirmed” in ONS when we define an ONS IPF death as one in which IPF is listed anywhere on the death certificate (as opposed to the underlying cause), we adopted the former definition as our gold standard.

The results of our second sensitivity analysis, in which we assessed the concordance of extending our HES-APC case definition to include patients who had episodes of hospital care in which IPF was listed further down the list of clinical diagnoses (beyond the first and second positions) impacted the diagnostic accuracy quite significantly. When we included patients with IPF codes (J84.1, J84.8 or J.84.9) in the top three diagnostic positions the size of the HES-APC cohort increased from 10,714 to 13,957, but the proportion of patients who also had a record in ONS decreased from 62.1 to 55.8% (Supplementary Fig. S3).

### Algorithm validation

The PPVs and sensitivities for our eight diagnostic algorithms are shown in Table 4.

**Table 4:** Positive predictive value (PPV) of code-based algorithms for IPF case finding in routinely-collected health data

Diagnostic algorithm	Data source	Description	No. of patients found	No. of patients meeting gold standard	PPV (95% CI)	Sensitivity (95% CI)
DA1	Aurum	• Any broad code in Aurum	9,498	6,113	64.3% (63.3–65.3)	53.5% (52.9–54.1)
DA2	Aurum and HES DID	• Any broad code in Aurum and • Evidence of HRCT of thorax at any time and • No other known causes of ILD recorded in Aurum^a^	4,721	3,187	67.5% (66.1–68.8)	28.2% (27.3–29)
DA3	Aurum	• Any narrow code in Aurum	1,828	1,370	74.9% (72.8–76.9)	12.1% (11.5–12.7)
DA4	Aurum and HES DID	• Any narrow code in Aurum and • Evidence of HRCT of thorax at any time and • No other known causes of ILD recorded in Aurum^a^	891	706	79.2% (76.4–81.8)	6.3% (5.8–6.7)
DA5	HES-APC	• ICD-10 codes J84.1, J84.8 or J84.9 as primary or secondary discharge diagnosis in either the first or any subsequent “episodes” within a hospital spell	10,714	6,651	62.0% (61.1–63.0)	57.0 (56.4–57.7)
DA6	HES-APC and HES DID	• ICD-10 codes J84.1, J84.8 or J84.9 as primary or secondary discharge diagnosis in the first or any subsequent “episode” within a hospital spell and • Evidence of HRCT of thorax at any time	2,985	2,379	79.7% (78.2–81.1)	21.0% (20.3–21.8)
DA7	Aurum and HES-APC	• Any broad code in Aurum and • ICD-10 codes J84.1, J84.8 and J84.9 as primary or secondary discharge diagnosis in either the first or any subsequent “episodes” within a hospital spell	5,488	4,304	78.4% (77.3–79.5)	38.1% (37.2–39.0)
DA8	Aurum and HES-APC	• Any narrow code in Aurum and • ICD-10 codes J84.1, J84.8 or J84.9 as primary or secondary discharge diagnosis in the first or any subsequent “episode” within a hospital spell	1,190	974	81.8% (79.5–84.0)	8.6% (8.1–9.1)

*DA *Diagnostic algorithm, *IPF *Idiopathic pulmonary fibrosis, *ILD *Interstitial lung disease, *HRCT *High resolution computed tomography, *CPRD *Clinical Practice Research Datalink, *HES *Hospital Episode Statistics

^a^Connective tissue disease, pulmonary sarcoidosis, domestic and occupational environmental exposures, drug toxicity and other non-IPF ILD

#### Aurum code-based algorithms

Among the diagnostic algorithms based on a broad list of IPF codes (DA1 and DA2), there was little variation in the estimated PPVs. The PPV for DA1 was 64.3% (95% CI, 63.3–65.3) with a sensitivity of 53.5% (95% CI, 52.9–54.1). Refining the criteria, that is, restricting cases to people with no known causes of ILD and who also had evidence of a HRCT scan (DA2), had a modest effect on the PPV (PPV_DA2_=65.7%; 95% CI, 66.1–68.8%) but reduced the sensitivity to just 28.2% (95% CI, 27.3–29%).

The narrow-code-based algorithms (DA3 and DA4), produced notably higher PPVs compared with the corresponding broad-code-based algorithms (PPV_DA4_=74.2% vs. PPV_DA1_=64.3% but the gain in diagnostic accuracy is very much at the expense of sample size. Again, adding evidence of a HRCT scan and excluding other known causes of pulmonary fibrosis had a relatively small impact on diagnostic accuracy (PPV_DA5_=62.0%; vs. PPV_DA6_=79.7%), but a proportionally greater impact on the number of patients identified. Adding stricter case finding criteria halved the sample size from 1,828 to 891, reducing the sensitivity to under 10% (Table 4).

#### Aurum-plus-HES APC algorithms

The probability that a person with IPF codes in both Aurum and HES- APC (DA7) “truly” has IPF (measured against a death in ONS), was estimated to be 78.4% (95%CI; 77.3–79.5%), Using a narrow set of Aurum codes plus evidence of a hospitalisation for IPF gave the highest PPV of all eight algorithms (PPV_DA8_ =81.8%) but a sensitivity of just 8.6% (Table 4).

#### Demographic and clinical characteristics

Table 5 describes the demographic and clinical characteristics of a series of cohorts of IPF patients, defined using four different diagnostic algorithms, DA1, DA3, DA6 and DA7. The four patient cohorts are broadly similar both in terms of the demographic characteristics and the comorbidity profile, save for the slightly lower prevalence of comorbid COPD (and to a lesser extent asthma and heart failure) in the Aurum narrow cohort. Relative to values reported in the literature, median survival times were relatively low, ranging from 1.8 years (DA6) to 2.6 years (DA3).

**Table 5:** Demographic and clinical characteristics of IPF patient cohorts defined using selected code-based diagnostic algorithms

Characteristic	DA1 *N* = 9,498 % of total (n) unless otherwise stated	DA3 *N* = 3,449 % of total (n) unless otherwise stated	DA6 *N* = 2,069 % of total (n) unless otherwise stated	DA7 *N* = 5,488 % of total (n) unless otherwise stated
***Demographic***				
Median (IQR) age in years	77 (13)	76(14)	76(14)	76 (14)
≥ 65 years	86.2% (*n* = 8,191)	82.9% (*n* = 1,516)	84.4% (*n* = 2,522)	83.7% (*n* = 4,593)
Male	63.2% (*n* = 6,011)	63.8% (*n* = 661)	64.4% (1,922)	62.5% (*n* = 3,434)
Median (IQR) survival in years	2.0 (3.7)	2.6 (4.8)	1.8 (3.5)	2.2 (3.9)
Smoking history (ever/current)	66.2%(*n* = 6,288)	62.4%(*n* = 1,1420	68.5(2,046)	66.4% (*n* = 3,642)
***Comorbiditie*s**				
COPD	19.0% (*n* = 1,806)	14.7% (*n* = 269)	17.8% (*n *= 532)	16.1% (*n *= 886)
Asthma (in past 2 years)	10.1% (*n *= 959)	7.9% (*n *= 146)	8.9% (*n *= 268)	9.4% (*n *= 518)
Lung cancer	2.1% (*n *= 206)	1.5% (*n *= 28)	2.3% (*n *= 70)	1.6% (*n *= 85)
Ischemic stroke	12.0% (*n *= 1,142)	10.1% (*n *= 185)	12.4% (*n *= 370)	11.8% (*n *= 645)
Heart failure	17.1% (*n *= 1,629)	13.6% (*n *= 249)	16.3% (*n *= 489)	16.4% (*n *= 899)
Ischemic heart disease	13.6% (*n *= 1,300)	11.8% (*n *= 217)	14.4% (*n *= 430)	13.3% (*n *= 735)
Myocardial infarction	13.3% (*n *= 1,267)	11.2% (*n *= 206)	12.4% (*n *= 370)	12.7% (*n *= 693)
Gastroesophageal reflux	17.6% (*n *= 1,677)	15.8% (*n *= 289)	20% (*n *= 597)	18.2% (*n *= 1,002)

*DA *Diagnostic algorithm, *IQR *Interquartile range, *COPD *Chronic pulmonary obstructive disease

^a^ Additional criteria include patients ≥ 18 years at IPF diagnosis and evidence of HRCT of the thorax at any time and exclude any patients with other known causes of ILD recorded in Aurum

## Discussion

The primary aim of this study was to investigate the diagnostic accuracy of the recording of IPF in UK primary and secondary care records. The lowest PPV was achieved using an algorithm based on secondary care data only (PPV_DA6_=62.4% (95% CI: 61.1–63.0) and the highest by an algorithm based on a limited set of primary care clinical codes coupled with evidence of a hospitalisation for IPF (PPV_DA8_=to 81.8% (95%CI: 79.5–84.0). While the two IPF patient cohorts defined by these two algorithms do not differ significantly in terms of their demographic characteristics or comorbidity profiles, the former (the secondary care-based cohort) has a slightly lower median survival time, implying that patients with more advanced disease might be overrepresented in a cohort derived using this algorithm.

We have shown that use of a set of clinical diagnostic codes to find people with IPF from their primary care records alone is an acceptable strategy for most observational research purposes. Measured against evidence of death in which IPF is at least a contributory factor, our baseline algorithm (DA1) achieved a reasonable PPV of 64.4%. Restricting the code set to a smaller number of highly specific IPF diagnostic codes (DA3) increased the PPV to 74.9%, similar to other conditions [10, 17]. However, the sizeable improvement in PPV achieved by limiting codes significantly reduced the sample size (9,498 vs. 1,828 individuals).

Attempts to improve diagnostic accuracy of our baseline broad and narrow clinical code-based algorithms by adding additional criteria produced only marginal improvements in the PPVs but had a significant impact on sensitivity. Adding evidence of a hospital admission had a relatively greater impact on the PPV of our baseline algorithms but without compromising sample size to the same extent. Therefore, we would recommend, that algorithms based on a narrow set of highly specific IPF clinical codes would be appropriate for studies which demand high case validity. For studies where sample size is a primary consideration, and certainly for estimating IPF incidence and prevalence, use of a broader code list combined with evidence of a hospitalisation represents a good compromise between acceptable case validity and capture (DA7).

A relatively high validity of a positive IPF diagnosis in primary care is not totally unexpected, even though most GPs will have limited experience of IPF [11]. It is unlikely that patients would be coded as having IPF by their GP without a prior referral to a pulmonary specialist and completion of confirmatory investigations, and therefore patients with an IPF diagnostic code in Aurum, especially one of the definitive “narrow” IPF codes, will likely truly have the disease. Support for this comes from a piece of qualitative research which revealed a general reluctance among GPs to code a condition that initially presents with non-specific symptoms without confirmatory evidence of the precise diagnosis [18]. The reluctance of GPs to assign an IPF code until they are sure of the diagnosis may also explain the relatively short median survival time (c 2 years from diagnosis) that we observed in our primary care study cohort.

The relatively high baseline PPV of a hospitalisation for IPF relative to a record in CPRD Aurum (62% v 64%) against an ONS death was consistent with earlier research which suggests that a high proportion of hospitalised patients who were assigned an IPF diagnostic code did indeed have the disease (c. 80%) [19]. However, this was however a small study, limited to four hospitals, and predated changes in IPF disease nomenclature and the use of ICD-10 codes, and thus may not reflect current coding practice. Furthermore, as our three-way concordance analysis showed, the majority of patients who died of or with IPF (58.9*%*) had been hospitalised for IPF prior to their death.

We noted some improvement in the PPVs for algorithms which included additional information relative to our baseline clinical-code-only algorithms, but this was relatively modest. This mirrors findings of other validation studies, including one for COPD that likewise reported that requiring evidence of spirometry and/or COPD medications only marginally improved the accuracy of simple diagnostic code-based algorithms (89.4% vs. 87.5% vs. 86.5%) [20]. Other studies which have assessed the relative merits of using supporting evidence to improve IPF case ascertainment have tended to approach this from the perspective of claims data and large health-related administrative databases that rely on ICD-10 coding but have reached similar conclusions [21, 22]. For most studies and settings, these marginal benefits will need to be weighed up against the often, large negative impact on sample size. This was particularly evident in this study, where we found that among our IPF cohorts, only a small proportion, had evidence of thoracic imaging in HES DID data. The other factor to consider when deciding on whether to use supporting evidence is data accessibility and cost; requests for HES DID data for example incur not inconsiderable cost.

Over the period of this study, we observed shifts in code use frequencies, most noticeably a gradual decline in the use of codes associated with now obsolete terms such as “cryptogenic fibrosing alveolitis” and a corresponding increase in codes associated with the term “idiopathic pulmonary fibrosis”. It is important to be aware of such trends when selecting appropriate codes for IPF case finding, especially for studies which cover a 10-year-plus time span. While changes in code frequencies related to changes in nomenclature are easier to rationalise, it is likely that some code shifts are being driven by more subtle or nuanced changes in GP coding practice that are harder to predict. Anecdotal evidence suggests that in the UK some GPs select a more generic code to document a given condition, especially if that code appears at the top of a drop-down menu. The shift towards the use of more specific codes for IPF might be a consequence of the introduction of antifibrotics and a desire of GPs to ensure that their patients met the eligibility criteria for prescription of an antifibrotic.

This study has several strengths and limitations. Its main strength stems from the use of a large population-based, representative healthcare datasets. Secondly, by selecting a study period which spans the period 2008–2018, we have been able to assess whether there have been any changes in coding practices over time, in the wake of the introduction and adoption of updated international IPF guidelines in 2011/18and the licensed use of anti-fibrotic drugs [1, 9]. The increased use of IPF-specific codes at the expense of now obsolete terms such as cryptogenic alveolitis and more generic codes such as diffuse pulmonary fibrosis after 2012 alludes to attempts to standardise how IPF is diagnosed and recorded in primary care. The main weakness of this study stems from the limitations of our chosen gold standard IPF population. While we acknowledge that a death in ONS is not a perfect gold standard, it was selected in the absence of viable, cost-effective alternatives. The main limitations of ONS as a gold standard include incompleteness; not everyone with IPF will necessarily die of IPF or have it recorded on their death certificate, especially if IPF was a not contributory factor in their death. However, the fact that a large proportion of people who had a GP-recorded IPF diagnosis but no mention of IPF on their certificate died of a respiratory reason (e.g. pneumonia), suggests that this group of patients did in all likelihood have IPF and do represent IPF-related deaths despite the absence of IPF in their death certificate. Moreover, by choosing a gold standard population that included people who had IPF mentioned anywhere on their death certificate as opposed to a population limited to those in whom IPF was recorded as the underlying cause, we were able to capture not only those who died of IPF but also those who died with IPF.

We also acknowledge that our recommended approach to case finding likely risks including some cases that have a non-IPF fibrotic ILD. Given a tendency among some healthcare practitioners noted previously to choose less specific codes, this is to a certain extent inevitable and represents a further limitation. We would advise researchers whose study question requires a highly specific IPF study population to also consider excluding people who have known causes of pulmonary fibrosis, such as a connective tissue disease.

## Conclusion

This is the first study to validate the completeness and quality of the recording of IPF in routinely-collected health data from the UK. It is important to conduct studies of this type and ensure the reliability of coding and case finding in UK electronic health records, given that these data are used to estimate disease prevalence and thus inform health service planning and resource allocation. More specifically, this work has demonstrated that for many observational research purposes, it is acceptable/feasible to use a simple set of clinical codes to define a cohort of patients with IPF in primary care data alone. However, those wishing to conduct observational research using UK health data are advised to tailor their choice of individual codes to their particular research objectives and study design and to consider the relative merits of using linked hospital data to improve diagnostic accuracy of a primary care study population.

## Supplementary Information

**Additional file 1.**

## Data Availability

Data are available on request from the CPRD. Their provision requires the purchase of a license, and this license does not permit the authors to make them publicly available to all. This work used data from the version collected in November 2020 and have clearly specified the data selected in the Methods section. To allow identical data to be obtained by others, via the purchase of a license, the code lists have been provided on GitHub. Licenses are available from the CPRD: The Clinical Practice Research Datalink Group, The Medicines and Healthcare products Regulatory Agency, 10 South Colonnade, Canary Wharf, London E14 4PU. This study used existing data from the UK CPRD electronic health record database, this data resource is accessible only to researchers with protocols approved by the CPRD’s independent scientific advisory committee; therefore, no additional unpublished data are available. All data management and analysis computer code are available on request (j.quint@imperial.ac.uk).
