# Supplementary material for: Validation of the recording of idiopathic pulmonary fibrosis in routinely collected electronic healthcare records in England
Source: BMC Pulm Med. 2023 Jul 11;23:256. doi: 10.1186/s12890-023-02550-0 (PMC10337174; doi:10.1186/s12890-023-02550-0)
Supplement: Supplementary file 1 — Additional file 1. [file 12890_2023_2550_MOESM1_ESM.docx]

**Supplementary material**

Validation of the recording of Idiopathic Pulmonary Fibrosis in routinely-collected electronic healthcare records in England

Dr Ann Morgan, Rikisha Shah Gupta, Dr Peter M George, Prof. Jennifer K Quint

Corresponding author: Jennifer Quint j.quint@imperial.ac.uk

File name: supplementary material.pdf

Note: this material has not been peer reviewed.

**Table S1**: IPF codes: CPRD Aurum medcodes, Read codes and SNOmed CT codes

| **Medcodeid (Aurum code identifier)** | **Read code** | **SnomedCT concept id** | **SnomedCT description id** | **Term** | **Narrow** | **Broad** |
| --- | --- | --- | --- | --- | --- | --- |
| 909731000006117 |  | 909731000006101 | 909731000006117 | [rfc] pulmonary fibrosis | 0 | 1 |
| 196654011 | H563.12 | 700250006 | 2989718018 | Cryptogenic fibrosing alveolitis | 1 | 1 |
| 5054601000006119 |  | 233703007 | 3037993016 | Diffuse parenchymal lung disease | 0 | 1 |
| 621121000006112 | H563100 | 196125002 | 301706014 | Diffuse pulmonary fibrosis | 0 | 1 |
| 4782331000006111 |  | 196125002 | 301707017 | DIPF - Diffuse interstitial pulmonary fibrosis | 0 | 1 |
| 4782341000006118 |  | 196125002 | 301708010 | Fibrosing alveolitis | 0 | 1 |
| 3336391000006117 |  | 51615001 | 85957013 | Fibrosis of lung | 0 | 1 |
| 815341000006115 | H563.11 | 236302005 | 2579204016 | Hamman - Rich syndrome | 0 | 1 |
| 885481000006119 | H563.99 | 45157009 | 885481000006119 | idiopath. fibrosing alveolitis | 1 | 1 |
| 12757781000006113 |  | 45157009 | 75302014 | idiopathic fibrosing alveolitis | 1 | 1 |
| 75302014 | H563.00 | 700250006 | 2989716019 | Idiopathic fibrosing alveolitis | 1 | 1 |
| 301709019 | H563z00 | 700250006 | 2989716019 | Idiopathic fibrosing alveolitis NOS | 1 | 1 |
| 12716191000006115 |  | 45157009 | 197847012 | idiopathic interstitial pneumonia | 0 | 1 |
| 1786131000006116 | H563.13 | 700250006 | 2989760019 | Idiopathic pulmonary fibrosis | 1 | 1 |
| 5054591000006110 |  | 233703007 | 350186017 | ILD - Interstitial lung disease | 0 | 1 |
| 107470010 | H56y100 | 64667001 | 107470010 | Interstitial pneumonia | 0 | 1 |
| 3551861000006113 |  | 64667001 | 107472019 | Interstitial pneumonitis | 0 | 1 |
| 254002014 | 23E5.11 | 162974009 | 254002014 | O/E - fibrosis of lung | 0 | 1 |
| 254003016 | 23E5.00 | 162974009 | 254003016 | O/E - fibrosis of lung present | 0 | 1 |
| 4555561000006111 |  | 162974009 | 2668607013 | On examination - fibrosis of lung present | 0 | 1 |
| 5492481000006115 |  | 266368002 | 3035384014 | Post inflammatory pulmonary fibrosis | 0 | 1 |
| 350255018 | H48..00 | 233749003 | 350255018 | Progressive massive fibrosis | 0 | 1 |
| 85960018 | H563200 | 51615001 | 85960018 | Pulmonary fibrosis | 0 | 1 |
| 885491000006116 | H563199 | 51615001 | 885491000006116 | Pulmonary fibrosis | 0 | 1 |
| 4782361000006119 |  | 196125002 | 2475607017 | UIP - Usual interstitial pneumonitis | 1 | 1 |
| 7532701000006115 |  | 700250006 | 2989702012 | Usual interstitial pneumonia | 1 | 1 |
| 2475606014 | H563300 | 196125002 | 2475606014 | Usual interstitial pneumonitis | 1 | 1 |

**Table S2.** Frequency of use of IPF clinical codes^a^ in UK primary care (CPRD Aurum data set), 2008–2018

| Snomed Concept ID | Description | Frequency of codes (n) | Frequency of codes (%) |
| --- | --- | --- | --- |
| 45157009 | idiopath. fibrosing alveolitis | 251 | 0.3% |
| 51615001 | Pulmonary fibrosis ; fibrosis of lung | 21720 | 25.3% |
| 64667001 | Interstitial pneumonia | 1535 | 1.8% |
| 162974009 | O/E - fibrosis of lung ; O/E - fibrosis of lung present | 4570 | 5.3% |
| 196125002 | Diffuse pulmonary fibrosis; Ususal interstitial pneumonitis | 32951 | 38.4% |
| 233749003 | Progressive massive fibrosis | 77 | 0.1% |
| 236302005 | Hamman - Rich syndrome | 10 | 0.0% |
| 700250006 | Idiopathic pulmonary fibrosis | 19826 | 23.1% |
| 909731000006101 | [rfc] pulmonary fibrosis | 4789 | 5.6% |

**Figure S1**. Patient attrition in three data sets: CPRD Aurum, HES-APC and ONS


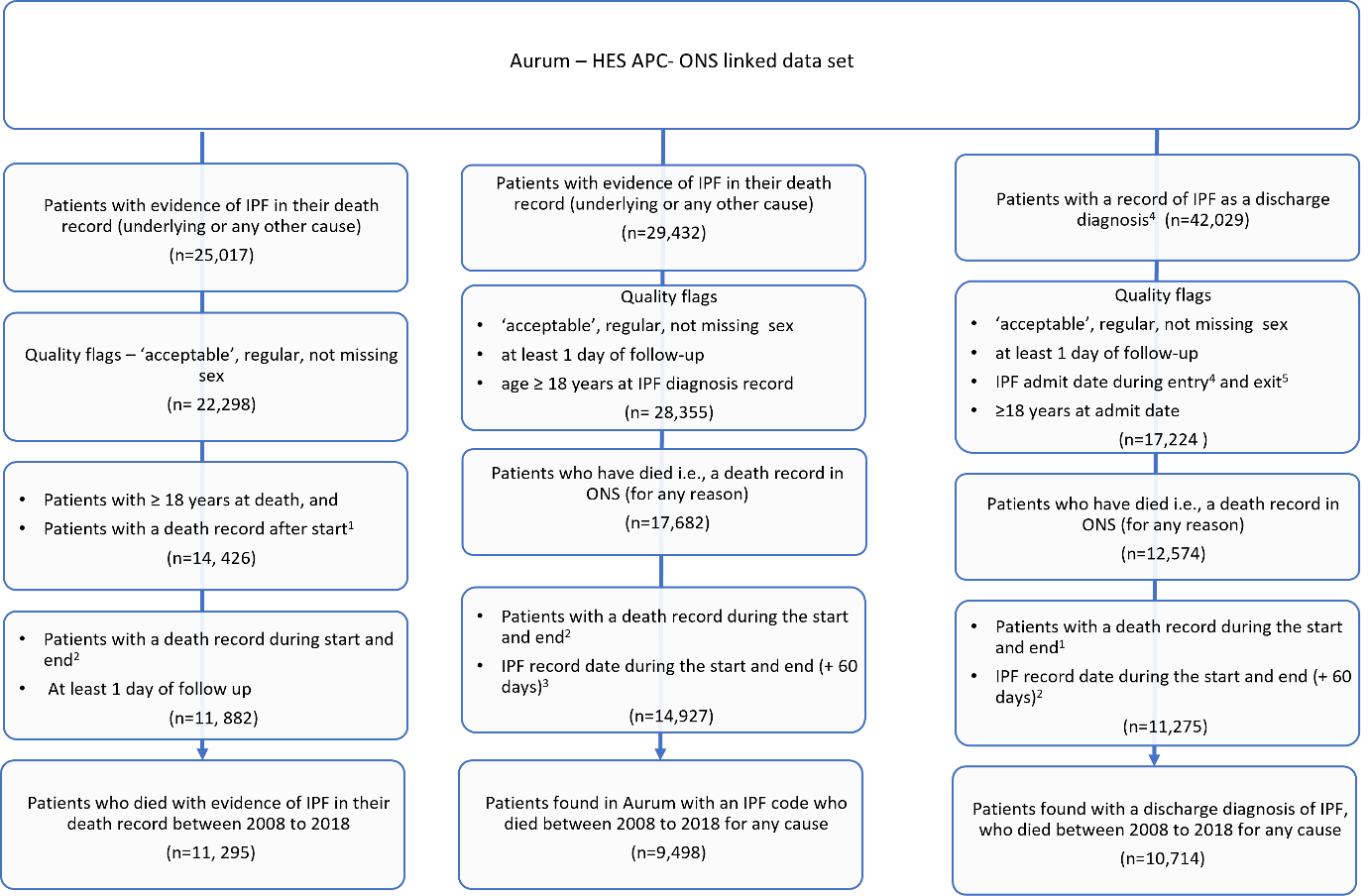


^1^ Start is defined as the latest of the start of the study period, registration start date, or date of 18^th^ birthday.

^2^ End is defined as the earliest end of the study period, last data collection date, or registration end date.

^3^ A 60-days grace period was used to allow an IPF record to be captured in Aurum.

^4^ Entry is defined as the latest of the registration start date, or the date of 18^th^ birthday..

^5^ Exit is defined as earliest of last data collection date, or registration end date.

**Figure S2.** Sensitivity analysis: effect of restricting the ONS cohort to those who had IPF coded as the underlying cause of death in the concordance in the recording of IPF between ONS (underlying cause of death as IPF), Aurum and HES APC (restricted to patients who died during the study period 2008–2018)


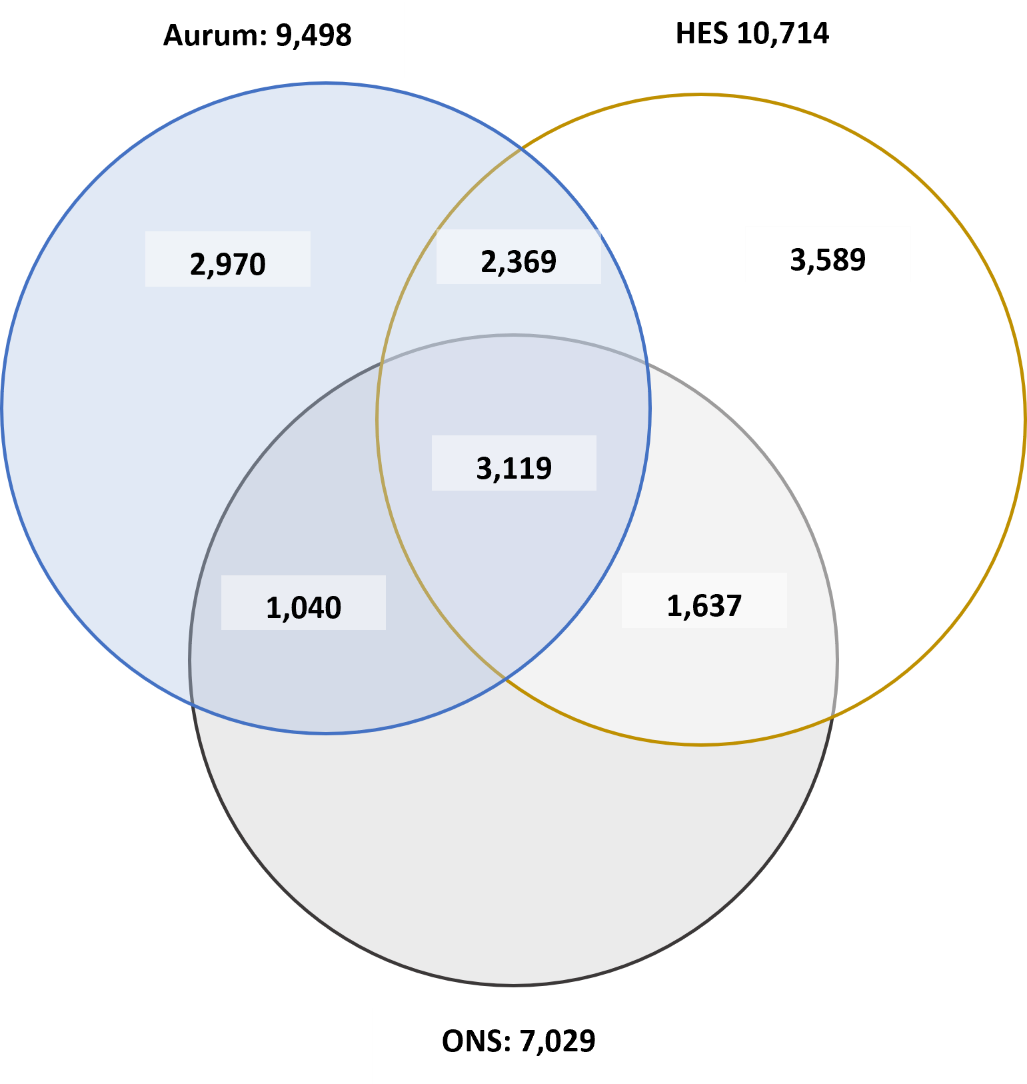


ONS, study eligible population with IPF as the underlying cause of death in their death record during the study period; Aurum, patients with one clinical code denoting IPF in Aurum (using a broad set of codes) and had died for any reason during the study period; HES-APC, patients admitted to the hospital for IPF (primary and secondary discharge diagnosis of IPF) at some point prior to their death for any reason (during the study period).

**Figure S3.** Sensitivity analysis: effect of extending the HES APC cohort to include those who had IPF listed in the third diagnostic position on the concordance in the recording of IPF between ONS, Aurum and HES APC (restricted to patients who died during the study period 2008–2018)


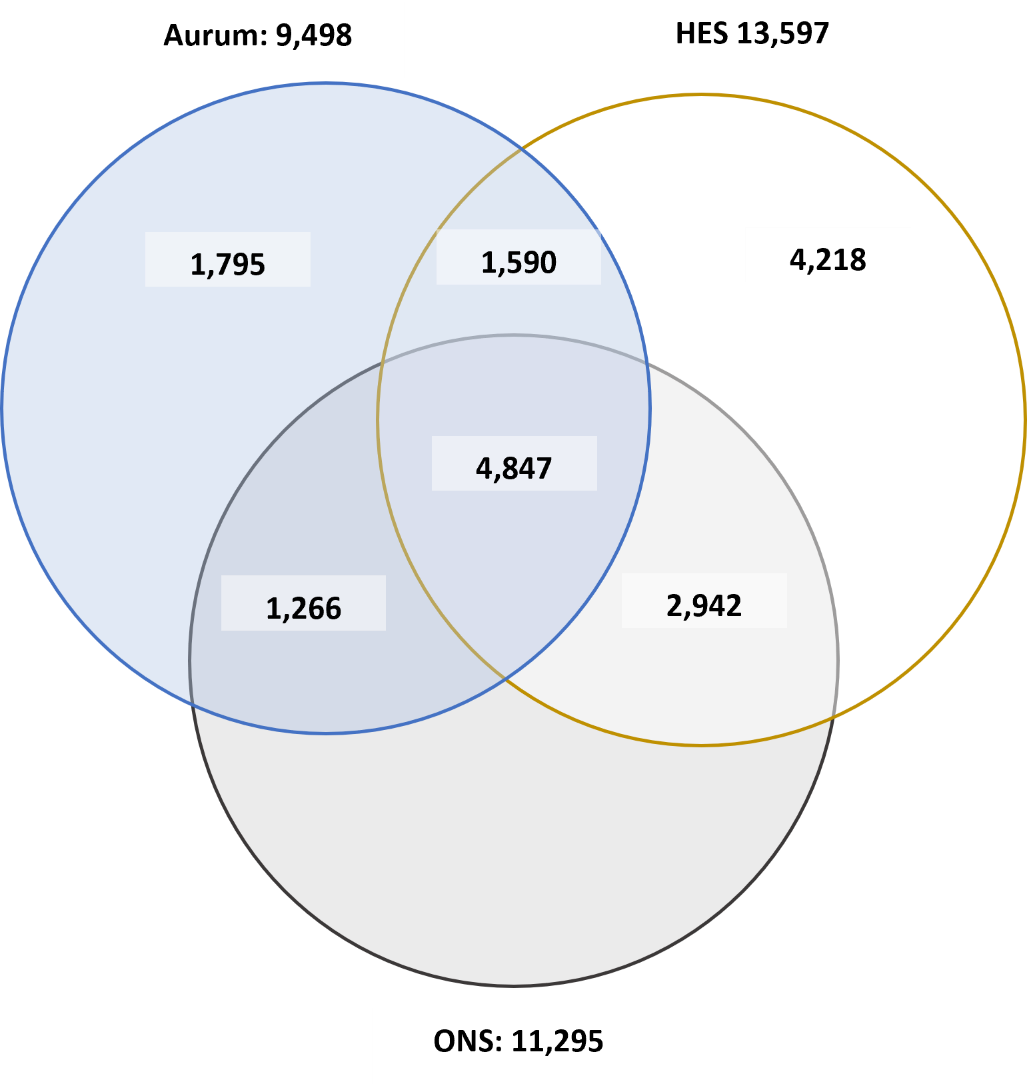


ONS, study eligible population with IPF as the underlying cause of death in their death record during the study period; Aurum, patients with one clinical code denoting IPF in Aurum (using a broad set of codes) and had died for any reason during the study period; HES-APC, patients admitted to the hospital for IPF (primary or any subsequent discharge diagnosis of IPF) at some point prior to their death for any reason (during the study period).
